# Robotic Versus Laparoscopic Gastrectomy for Gastric Cancer: A Mega Meta-Analysis

**DOI:** 10.3389/fsurg.2022.895976

**Published:** 2022-06-28

**Authors:** Shantanu Baral, Mubeen Hussein Arawker, Qiannan Sun, Mingrui Jiang, Liuhua Wang, Yong Wang, Muhammad Ali, Daorong Wang

**Affiliations:** ^1^ Clinical Medical College, Yangzhou University, Yangzhou China; ^2^ Department of Gastrointestinal Surgery, Northern Jiangsu People’s Hospital, Yangzhou China; ^3^ General Surgery Institute of Yangzhou, Yangzhou University, Yangzhou China; ^4^ Yangzhou Key Laboratory of Basic and Clinical Transformation of Digestive and Metabolic Diseases, Yangzhou China

**Keywords:** robotic, laparoscopic, gastrectomy, gastric cancer, meta-analysis

## Abstract

**Background:**

Laparoscopic gastrectomy and robotic gastrectomy are the most widely adopted treatment of choice for gastric cancer. To systematically assess the safety and effectiveness of robotic gastrectomy for gastric cancer, we carried out a systematic review and meta-analysis on short-term and long-term outcomes of robotic gastrectomy.

**Methods:**

In order to find relevant studies on the efficacy and safety of robotic gastrectomy (RG) and laparoscopic gastrectomy (LG) in the treatment of gastric cancer, numerous medical databases including PubMed, Medline, Cochrane Library, Embase, Google Scholar, and China Journal Full-text Database (CNKI) were consulted, and Chinese and English studies on the efficacy and safety of RG and LG in the treatment of gastric cancer published from 2012 to 2022 were screened according to inclusion and exclusion criteria, and a meta-analysis was conducted using RevMan 5.4 software.

**Results:**

The meta-analysis inlcuded 48 literatures, with 20,151 gastric cancer patients, including 6,175 in the RG group and 13,976 in the LG group, respectively. Results of our meta-analysis showed that RG group had prololonged operative time (*WMD *= 35.72, 95% *CI *= 28.59–42.86, *P *< 0.05) (RG: mean ± SD = 258.69 min ± 32.98; LG: mean ± SD = 221.85 min ± 31.18), reduced blood loss (*WMD* = −21.93, 95% *CI* = −28.94 to −14.91, *P *< 0.05) (RG: mean ± SD = 105.22 ml ± 62.79; LG: mean ± SD = 127.34 ml ± 79.62), higher number of harvested lymph nodes (*WMD *= 2.81, 95% *CI* = 1.99–3.63, *P *< 0.05) (RG: mean ± SD = 35.88 ± 4.14; LG: mean ± SD = 32.73 ± 4.67), time to first postoperative food intake shortened (*WMD* = −0.20, 95% *CI* = −0.29 to −0.10, *P* < 0.05) (RG: mean ± SD = 4.5 d ± 1.94; LG: mean ± SD = 4.7 d ± 1.54), and lower length of postoperative hospital stay (*WMD* = −0.54, 95% *CI* = −0.83 to −0.24, *P *< 0.05) (RG: mean ± SD = 8.91 d ± 6.13; LG: mean ± SD = 9.61 d ± 7.74) in comparison to the LG group. While the other variables, for example, time to first postoperative flatus, postoperative complications, proximal and distal mar gin, *R*_0_ resection rate, mortality rate, conversion rate, and 3-year overall survival rate were all found to be statistically similar at *P *> 0.05.

**Conclusions:**

In the treatment of gastric cancer, robotic gastrectomy is a safe and effective procedure that has both short- and long-term effects. To properly evaluate the advantages of robotic surgery in gastric cancer, more randomised controlled studies with rigorous research methodologies are needed.

## Introduction

Gastric cancer is the most common malignant tumor of the digestive tract in far-eastern countries. The global incidence of gastric cancer has declined steadily in recent years, but Asia still has the highest incidence of gastric cancer (1). Due to the lack of early diagnosis methods, most patients are already in the middle and late stages of the disease at the time of their diagnosis. The best method of treatment is currently surgery. The surgical method has evolved from traditional open surgery to laparoscopic surgery (2). Since the mid-1980s, laparoscopic techniques have received increasing recognition for their minimally invasive advantages in treating gastric cancer (3), and laparoscopic gastrectomy (LG) has become the standard treatment for early gastric cancer. Nonetheless, laparoscopic techniques have some limitations and shortcomings, including inflexible operation of surgical instruments, two-dimensional imaging display interface, and a limited range of operation. In recent years, robotic technologies have made tremendous progress in overcoming the technological limitations of traditional laparoscopy. Robot-assisted surgical procedure has visual direction from the bottom to the top and not the other way around as in traditional open surgery, which makes it more advantageous to expose the dirty surface tissue. Although several scholars have conducted meta-analyses of such studies, all of them focused on assessing its immediate efficacy without considering its long-term effectiveness, such as its 3-year survival rate, and some of the results differed from study to study. As the robotic surgery system continues to advance, both its technology and efficacy are continually improving, and the research reports associated with it continue to be updated. In addition, the robotic system was only recently applied to some patients undergoing gastric cancer surgery, and its status in the treatment of gastric cancer has not been conclusively established or included in guidelines. To evaluate the short and long-term efficacy and safety of the robotic surgery system in the clinical treatment of gastric cancer, this study conducted a meta-analysis of published clinical comparative studies (3–31) on RG and LG.

## Materials & Methods

### Search Strategy

In order to search PubMed, Medline, Cochrane Library, Embase, Google Scholar, and China Journal Full-text Database (CNKI) and other databases according to clinical comparison studies of RG and LG, the search strings “Robotic OR da Vinci OR Robot-Assisted”, “Gastrectomy”, “Gastric “, “Cancer OR Carcinoma OR Tumor OR Neoplasm”, “Laparoscopic OR Laparoscopic-Assisted “ and “Robotic”, Searches were limited to the period 2012–2021, with the “related search” feature being utilized further to exclude omissions.

### Literature Inclusion and Exclusion Criteria

Inclusion criteria: (1) published randomized or non-randomized controlled trials comparing RG with LG; (2) patients diagnosed with gastric cancer who have undergone their first surgical procedure; (3) provide clear criteria for the selection of study cases and methods for grouping; (4) provide evidence of clinical efficacy comparison between RG and LG; (5) include data studies of superior quality and detail; (6) describe the raw data, including continuous variables such as mean and standard deviation, and count information such as the number of events and the number of samples. For dichotomous variables, the combined odds ratio (OR) and 95% confidence interval (CI) should be provided, as well as a regression coefficient that can be converted to the combined OR and 95% CI and standard error. Exclusion criteria: (1) comparisons of non-LG and RG cases; (2) study cases containing other benign gastrointestinal diseases; (3) study cases having only undergone palliative major gastrectomy, tumor reduction, or short-circuit surgery; (4) study cases involving emergency surgery; (5) no reliable comparisons could be drawn from the literature; (6) duplicate published studies; (7) no controlled studies conducted simultaneously; and (8) no clear grouping tendency in terms of the extent of lymph node dissection or stage of the disease.

### Data Extraction

Extractions are made by two investigators independently, and if a dispute occurs, it is resolved through mutual discussion or by a third party. The following data types can be identified: (1) General information, including the names of the authors, the dates of literature publication, the type of study, the sample size, the tumor site and its size, and the TNM stage; (2) outcome indicators, such as operative time and blood loss, lymph node dissection, transit rate, distal margin length, R_0_ resection rate, postoperative hospital stay, immediate postoperative gas and food intake, complication rate, 3-year survival rate, and morbidity and mortality rate.

### Evaluation of the Quality of the Literature

We used the MINORS scoring criteria (32) to assess the quality of the clinical trials (score 0: no description, score 1: inadequate description, and score 2: adequate description). A modified set of MINORS scoring criteria containing 12 items, which yields scores ranging from 0 to 24 was used to evaluate the quality of the literature included in this study (Supplementary file Appendix 1).

### Statistical Analysis

We performed the meta-analysis using RevMan 5.4 software, using odds ratio (OR) values for measurement data and weighted mean differences (*WMD*) for efficacy analysis for count data. The 95% confidence interval (*CI*) for the effect sizes was calculated. It was checked for heterogeneity between the studies using the *χ*^2^ test and *I*^2^ values, and in case of heterogeneity (*I*^2^ > 50%, *P *< 0.05), a random-effects model was applied; if there was no heterogeneity (*I*^2^*^ ^*< 50%, *P*≥ 0.05), a fixed-effects model was applied. The differences were considered statistically significant at *P *< 0.05.

## Results

### Search Results

A preliminary search retrieved a total of 5,440 articles published from 2012 to 2021. After reviewing all titles and abstracts, 76 complete articles were found, 28 of which were rejected because they did not meet the inclusion criterion. Supplementary Figure 1 illustrates the search process. Ultimately, 20,151 patients data from 48 retrospective studies were included in the present study, with 6,175 in the RG group and 13,976 in the LG group (3–30). Table 1 presents the basic characteristics of the included literature and MINORS scale for quality assessment, while Table 2 provides the patients' characteristics of the included literature. Supplementary Figure 2 depicts MINORS scores bar graph for the observational studies included in our systematic review.

**Table 1:** Basic characteristics of the literatures included in the meta-analysis.

Author	Year	Country	Study period	Study design	Sample size		Surgical extension	Level of LND	MINORS
					RG	LG			
Eom (4)	2012	Korea	2009–2010	OCS (P)	30	62	D	D1, D2	22
Kang (5)	2012	Korea	2008–2011	OCS (P)	100	282	D, T	D1, D2	22
Yoon (6)	2012	Korea	2009–2011	OCS (R)	36	65	T	D1, D2	23
Uyama (7)	2012	Japan	2009–2010	OCS (P)	25	225	D	D2	21
Kim KM (8)	2012	Korea	2005–2010	OCS (P)	436	861	D, T	D1, D2	23
Huang (9)	2012	Taiwan	2006–2012	OCS (R)	39	64	D, P, T	D1, D2	22
Zhang XL (10)	2012	China	2009–2011	OCS (P)	97	70	D, P, T	D2	18
Hyun (11)	2013	Korea	2009–2010	OCS (P)	38	83	D, T	D1, D2	22
Kim HI (12)	2014	Korea	2003–2009	OCS (P)	172	481	D, T	D1, D2	22
Noshiro (13)	2014	Japan	2010–2012	OCS (P)	21	160	D	D1, D2	22
Huang (14)	2014	Taiwan	2008–2014	OCS (P)	72	73	D, T	D1, D2	22
Son T (15)	2014	Korea	2003–2010	OCS (P)	51	58	T	D2	22
Zhou (3)	2014	China	2010–2013	OCS (R)	120	394	D, P, T	D1, D2	23
Liu J (16)	2014	China	2012–2013	OCS (R)	100	100	D, P, T	D2	19
Han (17)	2015	Korea	2008–2013	OCS (R)	68	68	PPG	D1	23
Seo (18)	2015	Korea	2004–2009	OCS (P)	40	40	D	D1, D2	20
Park (19)	2015	Korea	2009–2011	OCS (P)	145	612	D, T	D1	19
Lee (20)	2015	Korea	2003–2010	OCS (P)	133	267	D	D2	21
Suda (21)	2015	Japan	2009–2012	OCS (R)	88	438	D, T	D1, D2	22
Shen (22)	2015	China	2011–2014	OCS (R)	93	330	D, T	D1, D2	21
Li P (23)	2015	China	2011–2014	OCS (R)	126	124	T	D2	21
Cianchi (24)	2016	Italy	2008–2015	OCS (P)	30	41	D	D1, D2	21
Kim HI (25)	2016	Korea	2011–2012	OCS (P)	185	185	D, T	D1, D2	23
Nakauchi (26)	2016	Japan	2009–2012	OCS (R)	84	437	D, T	D1, D2	23
Hong (27)	2016	Korea	2008–2015	OCS (P)	232	232	D	D1, D2	22
Kim YW (28)	2016	Korea	2009–2011	OCS (P)	87	288	D	D1, D2	20
Xue (29)	2016	China	2012–2014	OCS (R)	35	35	D	D2	20
Parisi (30)	2017	Italy	2015–2016	OCS (P)	151	151	D, T	D2	21
Yang (31)	2017	Korea	2009–2015	OCS (P)	173	511	D, T	D1, D2	21
Li GT (33)	2017	China	2017	OCS (R)	15	15	T	D2	20
Teng (34)	2017	China	2016–2017	OCS (R)	41	58	D	D1, D2	20
Hu (35)	2017	China	2014–2016	OCS (R)	39	39	D	D2	21
Lan (36)	2017	China	2014–2016	OCS (R)	196	673	D, P, T	NA	20
Liu HB (37)	2018	China	2017	OCS (R)	100	135	D, T	D1, D2	21
Lu (38)	2018	China	2016–2017	OCS (P)	101	303	D, T	D1, D2	20
Obama (39)	2018	Korea	2005–2009	OCS (P)	315	525	D, T	D1, D2	23
Zhang K (40)	2018	China	2011–2013	OCS (R)	27	62	D, P, T	D1	23
Li ZY (41)	2018	China	2013–2017	OCS (P)	112	112	D, T	D2	23
Li SY (42)	2018	China	2015–2017	OCS (R)	50	56	D	D2	21
Wang WJ (43)	2019	China	2016–2018	OCS (P)	251	276	D, T	D2	23
Gao (44)	2019	China	2011–2014	OCS (P)	163	339	D, P, T	D1, D2	21
Alhossaini (45)	2020	Korea	2015–2017	OCS (R)	25	30	T	NA	23
Ye SP (46)	2020	China	2014–2019	OCS (P)	285	285	D	D2	23
Shibasaki (47)	2020	Japan	2009–2019	OCS (P)	359	1042	D, P, T	D1, D2	22
Kong (48)	2020	China	2014–2017	OCS (R)	294	750	D, P, T	D1, D2	23
Shin (49)	2021	Japan	2009–2017	OCS (P)	421	1663	D, T, PPG	D1, D2	23
Hikage (50)	2021	Japan	2012–2020	OCS (P)	345	835	D, P, T	D1, D2	23
Li ZY (51)	2021	China	2006–2019	OCS (P)	29	41	D, P, T	NA	23

*NA, not available; OCS, observational clinical study; P, prospectively collected data; R, retrospectively collected data; D, distal gastrectomy; P, proximal gastrectomy; T, total gastrectomy; PPG, pylorus-preserving gastrectomy.*

**Table 2:** Patients’ characteristics of the included literature.

Author	Year	Gender (M/F)		Age		BMI (kg/m^2^)		TNM Stage
		RG	LG	RG	LG	RG	LG	
Eom (4)	2012	21/9	41/21	52.8 ± 11.5	57.9 ± 11	24.2 ± 4.5	24.1 ± 2.7	I, II, III
Kang (5)	2012	63/37	191/91	53.2 ± 12.03	58.78 ± 12.40	23.74 ± 3.72	23.63 ± 3.47	I, II, III
Yoon (6)	2012	18/18	31/34	53.9 ± 11.7	56.9 ± 12.3	23.2 ± 2.5	23.6 ± 3.4	T1∼3N0∼2
Uyama (7)	2012	14/11	156/69	61.6 ± 11.0	62.6 ± 9.9	22.6 ± 3.1	22.0 ± 3.1	T1N0
Kim KM (8)	2012	265/171	550/311	54.2 ± 12.5	58.8 ± 12.0	23.6 ± 3.1	23.5 ± 2.8	T0∼4N0∼3
Huang (9)	2012	19/20	43/21	65.1 ± 15.9	65.6 ± 14.8	24.2 ± 3.7	24.7 ± 3.3	I, II, III
Zhang XL (10)	2012	66/31	49/21	56.1 ± 5.8	54.8 ± 4.9	22.5 ± 3.6	21.7 ± 2.1	I, II, III
Hyun (11)	2013	25/13	55/28	54.2 ± 12.7	60.3 ± 12.3	23.8 ± 2.6	23.8 ± 2.9	I, II, III
Kim HI (12)	2014	103/69	294/187	55.2 13.0	61.3 ± 11.9	23.7 ± 2.9	23.6 ± 2.9	I, II, III
Noshiro (13)	2014	14/7	102/58	66 ± 10	69 ± 12	22.8 ± 3.1	21.8 ± 2.8	I, II, III, IV
Huang (14)	2014	40/32	42/31	67.7 ± 15.1	66.0 ± 13.5	24.1 ± 3.3	24.2 ± 3.3	I, II, III
Son T (15)	2014	23/28	36/22	55.3 ± 12.2	58.8 ± 12.2	22.7 ± 2.9	23.2 ± 3.3	I, II, III
Zhou (3)	2014	90/30	276/118	54.7 ± 10.1	55.6 ± 11.8	21.6 ± 2.8	21.7 ± 2.6	I, II, III
Liu J (16)	2014	59/41	63/37	66.4 ± 5.7	67.8 ± 4.8	22.4 ± 1.8	23.1 ± 1.2	I, II, III, IV
Han (17)	2015	31/37	32/36	50.6 ± 8.3	49.8 ± 11.5	22.7 ± 2.4	22.8 ± 3.0	I, II, III
Seo (18)	2015	19/21	20/20	51.6 ± 4.5	55.1 ± 5.1	23.6 ± 2.1	23.8 ± 1.9	I, II, III
Park (19)	2015	77/71	369/253	54.5 ± 11.6	58.3 ± 11.8	23.9 ± 3.3	23.9 ± 3.0	I, II, III
Lee (20)	2015	85/48	154/113	53.6 ± 13.2	59.2 ± 11.7	23.2 ± 2.7	23.7 ± 2.8	I, II, III
Suda (21)	2015	51/37	307/131	64 ± 13	68 ± 13.5	22.6 ± 3.9	21.8 ± 7.9	I, II, III, IV
Shen (22)	2015	75/18	249/81	56.8 ± 10.5	57.9 ± 11.5	24.3 ± 3.3	23.8 ± 3.6	I, II, III
Li P (23)	2015	70/56	64/60	56.7 ± 9.9	57 ± 10.6	21.4 ± 3.8	22.2 ± 3.7	NA
Cianchi (24)	2016	14/16	19/22	73 ± 10.2	74 ± 11.7	27 ± 3.7	26 ± 1.7	I, II, III
Kim HI (25)	2016	113/72	113/72	53.3 ± 11.4	56.0 ± 11.5	23.8 ± 3.0	23.6 ± 2.7	I, II, III
Nakauchi (26)	2016	48/36	307/130	64 ± 13	68 ± 13.5	22.6 ± 3.9	21.8 ± 5.2	NA
Hong (27)	2016	154/78	156/76	53.7 ± 11.5	55 ± 13.0	23.8 ± 3.3	23.8 ± 3.0	T1∼4N0∼3
Kim YW (28)	2016	46/41	170/118	54.1 ± 12.0	60.5 ± 11.0	24.1 ± 3.4	24.0 ± 4.3	I, II, III
Xue (29)	2016	26/9	25/10	59.2 ± 9.6	56.2 ± 14.1	24.6 ± 2.9	23.4 ± 2.3	I, II, III
Parisi (30)	2017	81/70	85/66	68.81 ± 12.12	65.82 ± 14.16	24.58 ± 3.00	24.02 ± 2.22	I, II, III
Yang (31)	2017	98/75	258/253	NA	NA	23.6 ± 3.2	23.7 ± 3.1	I, II, III
Li GT (33)	2017	14/1	10/5	58.73 ± 9.79	55.07 ± 14.07	22.42 ± 2.73	21.92 ± 3.39	I, II, III
Teng (34)	2017	29/12	40/18	58 ± 11.2	59 ± 9.8	24.25 ± 2.01	24.64 ± 2.80	I, II, III, IV
Hu (35)	2017	28/11	20/19	59. 41 ± 12. 34	56. 72 ± 12. 47	NA	NA	I, II, III
Lan (36)	2017	137/59	501/172	59 ± 11.6	59 ± 11.6	23.6 ± 4.6	23.5 ± 4.5	T0∼4N0∼3
Liu HB (37)	2018	79/21	101/34	58 ± 4.4	58 ± 3.7	21.2 ± 0.9	22 ± 1.0	I, II, III, IV
Lu (38)	2018	73/28	212/91	NA	NA	NA	NA	I, II, III
Obama (39)	2018	189/126	327/198	54.5 ± 12.6	59.3 ± 11.9	23.6 ± 3.1	23.5 ± 2.9	I, II, III
Zhang K (40)	2018	19/8	52/10	59.7 + 11.6	56.6 + 12.2	24.9 + 2.7	24.5 + 3.2	I, II, III
Li ZY (41)	2018	78/34	79/33	55.6 ± 11.3	56.1 ± 11.1	23.6 ± 2.9	23.6 ± 3.0	I, II, III
Li SY (42)	2018	35/15	39/17	65.6 ± 8.3	66 ± 7.4	24.3 ± 2.1	24.6 ± 2.4	T2, T3, T4a
Wang WJ (43)	2019	201/50	205/71	57.7 ± 11.2	56.8 ± 11.5	22.1 ± 3.5	22.4 ± 3.4	I, II, III
Gao (44)	2019	121/42	201/138	60.27 ± 10.50	59.36 ± 11.08	23.77 ± 3.11	23.44 ± 3.47	I, II, III
Alhossaini (45)	2020	17/8	22/8	54 ± 15	60 ± 15	22.5 ± 2.7	22.2 ± 2.9	I, II, III, IV
Ye SP (46)	2020	189/96	186/99	57.1 ± 8.3	57.0 ± 8.6	24.4 ± 2.3	24.5 ± 2.2	I, II, III
Shibasaki (47)	2020	233/126	740/302	67 ± 14.7	70 ± 17.2	22.8 ± 4.4	22.4 ± 5.6	I, II, III
Kong (48)	2020	221/73	536/214	58.57 ± 10.51	59.10 ± 10.20	22.9 ± 4.4	22.2 ± 5.7	I, II, III
Shin (49)	2021	264/157	1088/575	53 ± 12	60 ± 12	23.87 ± 3.13	23.89 ± 3.22	I, II, III
Hikage (50)	2021	219/126	595/240	67 ± 16	69 ± 16.5	22.3 ± 4.05	22.7 ± 5.5	I, II, III
Li ZY (51)	2021	22/7	31/10	60.3 ± 12.6	58.2 ± 9.8	19.4 ± 2.2	20.4 ± 2.5	I, II, III

*NA, not available; M male; F, female; RG, robotic gastrectomy; LG, laparoscopic gastrectomy.*

**Table 3:** Overall results of the meta-analysis.

Outcomes	No. of studies	Sample size		Heterogeneity		Overall effect size	95% *CI* of overall effect	*P* value
		RG	LG	*I*^2^ (%)	*P* value			
Operation time (min)	45	5900	13,199	97	<0.05	WMD = 35.72	28.59–42.86	<0.05
Intraoperative blood loss (mL)	43	5905	13,451	93	<0.05	WMD = −21.93	−28.94 to −14.91	<0.05
Lymph node dissection	46	5930	13,082	87	<0.05	WMD = 2.81	1.99–3.63	<0.05
Time to first flatus (days)	26	3084	5322	97	>0.05	WMD = −0.20	−0.42 to 0.02	>0.05
Time to first food intake (days)	26	3855	7160	53	<0.05	WMD = −0.20	−0.29 to −0.10	<0.05
Length of hospital stay (days)	46	6136	13,912	80	<0.05	WMD = −0.54	−0.83 to −0.24	<0.05
Postoperative complications	47	6136	13,937	22	>0.05	OR = 0.88	0.78–1.00	>0.05
Proximal margin (cm)	16	2176	4878	57	>0.05	WMD = −0.02	−0.23 to 0.19	>0.05
Distal margin (cm)	16	2125	4820	71	>0.05	WMD = 0.18	−0.71 to 0.48	>0.05
R_0_ resection rate	5	6175	13,976	0	>0.05	OR = 1.74	0.70–4.28	>0.05
Tumor size (cm)	22	3176	7295	95	>0.05	WMD = −0.19	−0.52 to 0.14	>0.05
Mortality rate	20	4239	9823	0	>0.05	OR = 1.16	0.76–1.76	>0.05
Conversion rate	14	3614	9773	0	>0.05	OR = 0.64	0.40–1.00	>0.05
Reoperation rate	13	2192	4693	0	>0.05	OR = 1.05	0.68–1.62	>0.05
Overall survival	12	1926	4857	88	>0.05	OR = 1.19	0.70–2.00	>0.05

### Meta-Analysis Results

**Figure 1:**
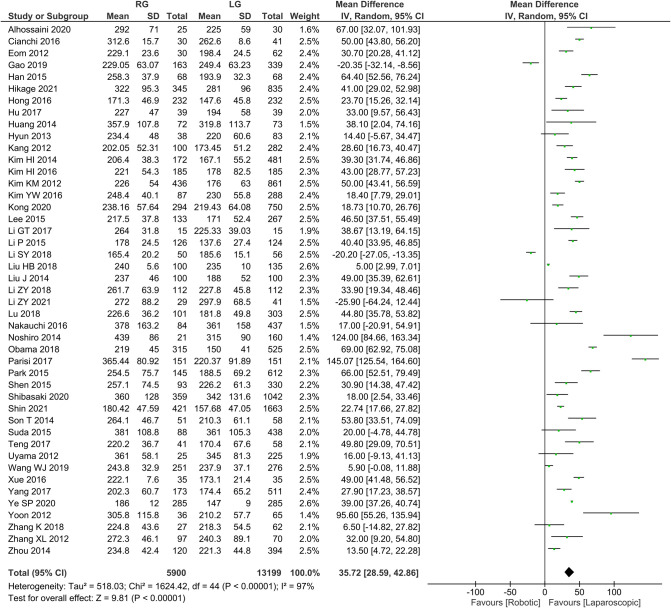
Comparison of operation time between RG and LG group.

Operation time was reported in 45 publications, with homogeneity test *I*^2 ^= 97%, *P *< 0.05. Using a random effect model analysis showed that the RG group had a significantly longer operation time than the LG group (*WMD *= 35.72, 95% *CI *= 28.59–42.86, *P *< 0.05) (Figure 1). The mean ± SD values are 258.69 min ± 32.98 and 221.85 min ± 31.18, for the RG and LG groups, respectively.

**Figure 2:**
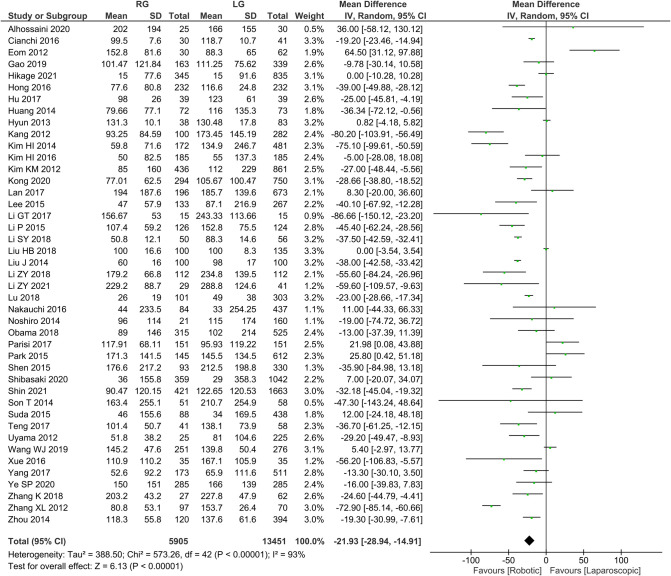
Comparison of intraoperative blood loss between RG and LG group.

Intraoperative bleeding was reported in 43 publications with homogeneity test *I*^2 ^= 93%, *P *< 0.05, and analysis using a random effects model showed that intraoperative bleeding was significantly less in the RG group than in the LG group (*WMD* = −21.93, 95% *CI* = −28.94 to −14.91, *P *< 0.05) (Figure 2). The mean ± SD values are 105.22 ml ± 62.79 and 127.34 ml ± 79.62, for the RG and LG groups, respectively.

**Figure 3:**
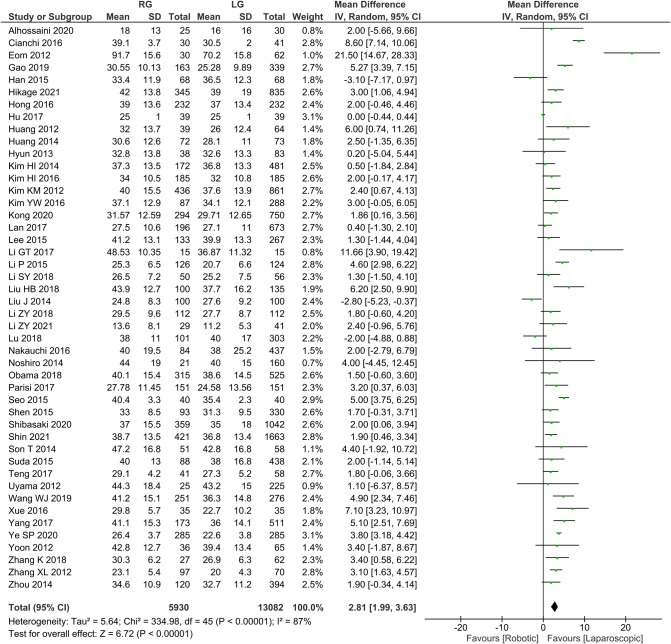
Comparison of the number of resected lymph nodes between RG and LG group.

Number of lymph node dissection 46 publications reported the number of lymph node dissections with homogeneity test *I*^2 ^= 87%, *P *< 0.05, and analysis using a random effects model showed that the number of lymph node dissections was higher in the RG group than in the LG group (*WMD *= 2.81, 95% *CI* = 1.99–3.63, *P *< 0.05) using random effects model analysis (Figure 3). The mean ± SD values are 35.88 ± 4.14 and 32.73 ± 4.67, for the RG and LG groups, respectively.

**Figure 4:**
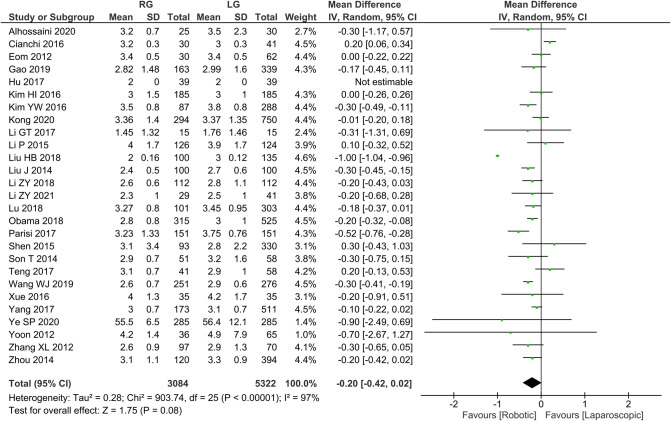
Comparison of time to first postoperative flatus between RG and LG group.

Time to first postoperative flatus 26 publications reported time to first postoperative flatus with homogeneity test *I*^2 ^= 97%, *P *< 0.05, and analysis using a random effects model showed not statistically significant in time to first postoperative flatus between the two groups (*WMD* = −0.20, 95% *CI* = −0.42 to 0.02, *P *> 0.05) (Figure 4). The mean ± SD values are 5.02 d ± 1.24 and 5.25 d ± 2.54, for the RG and LG groups, respectively.

**Figure 5:**
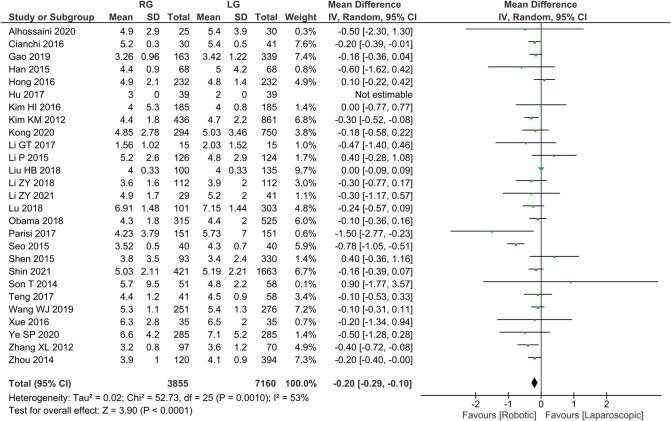
Comparison of time to first postoperative food intake between RG and LG group.

Time to first postoperative food intake 26 publications reported time to first postoperative food intake with homogeneity test *I*^2 ^= 53%, *P *< 0.05, and analysis using a random effects model showed that time to first postoperative food intake was significantly shorter in the RG group than in the LG group (*WMD* = −0.20, 95% *CI* = −0.29 to −0.10, *P* < 0.05) (Figure 5). The mean ± SD values are 4.55 d ± 1.94 and 4.76 d ± 1.54, for the RG and LG groups, respectively.

**Figure 6:**
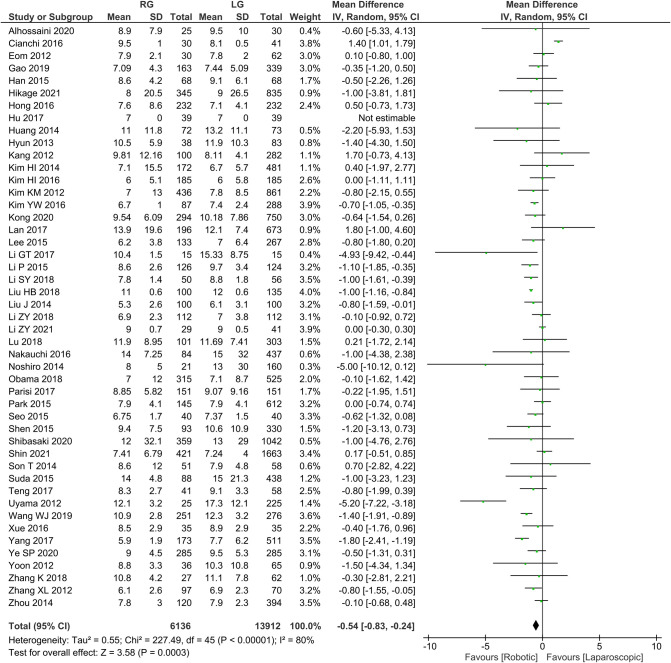
Comparison of length of postoperative hospital stay between RG and LG group.

Postoperative length of hospital stays 46 publications reported postoperative length of stay, homogeneity test *I*^2 ^= 80%, *P *< 0.05, and a random effects model analysis showed significantly lower length of hospital stay in the RG group than in the LG group (*WMD* = −0.54, 95% *CI* = −0.83 to −0.24, *P *< 0.05) (Figure 6). The mean ± SD values are 8.91 d ± 6.13 and 9.61 d ± 7.74, for the RG and LG groups, respectively.

**Figure 7:**
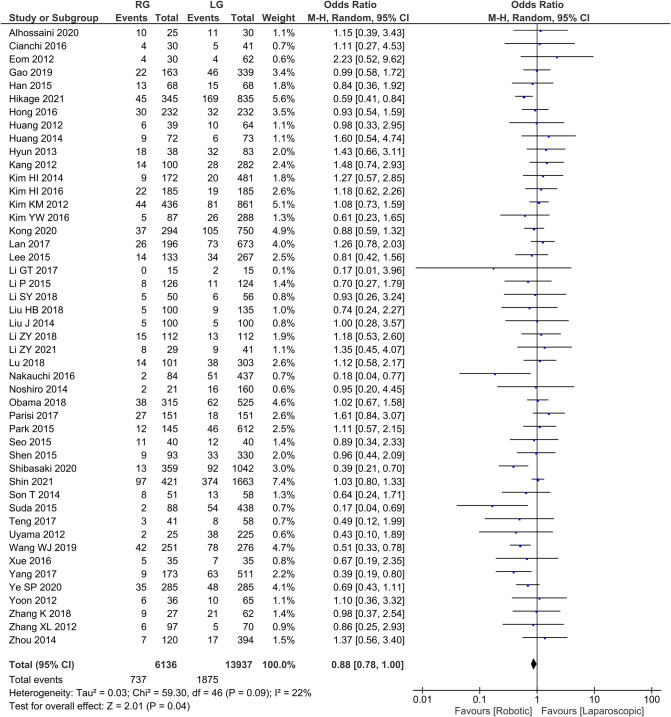
Comparison of postoperative complications between RG and LG group.

Postoperative Complication rates 47 publications reported complication rates with homogeneity test *I*^2 ^= 22%, *P *> 0.05, and a random effects model analysis showed no statistically significant difference in complication rates between the two groups (*OR *= 0.88, 95% *CI *= 0.78–1.00, *P *< 0.05) (Figure 7). The average complication rate was 15.68 in RG group and 39.89 in LG group.

Proximal margin distance 16 publications reported proximal margin distance with homogeneity test *I*^2 ^= 57%, *P *< 0.05, and analysis using a random effects model analysis showed no statistically significant difference in proximal margin distance between the two groups (*WMD* = −0.02, 95% *CI* = −0.23 to 0.19, *P *> 0.05) (Supplementary Figure 3). The mean ± SD values are 4.05 cm ± 1.15 and 4.05 cm ± 0.94, for the RG and LG groups, respectively.

Distal margin distance 16 publications reported distal margin distance with homogeneity test *I*^2 ^= 71%, *P *< 0.05, and a random effects model analysis showed not statistically significant in distal margin distance between the two groups (*WMD *= 0.18, 95% *CI* = −0.71 to 0.48, *P *> 0.05) (Supplementary Figure 4). The mean ± SD values are 5.98 cm ± 1.56 and 5.66 cm ± 1.89, for the RG and LG groups, respectively.

R_0_ resection rates 48 publications reported R_0_ resection rates with homogeneity test *I*^2 ^= 0%, *P *> 0.05, and analysis using a fixed effect model showed no statistically significant difference in R_0_ resection rates between the two groups (*OR *= 1.74, 95% *CI *= 0.70–4.28, *P *> 0.05) (Supplementary Figure 5). The average R_0_ resection rate was 128.52 in RG group and 290.81 in LG group.

Tumor size 22 publications reported tumor size with homogeneity test *I*^2 ^= 95%, *P *< 0.05, and analysis using a random effects model analysis showed no statistically significant difference in tumor size between the two groups (*WMD* = −0.19, 95% *CI* = −0.52 to 0.14, *P *> 0.05) (Supplementary Figure 6). The mean ± SD values are 3.27 cm ± 0.82 and 3.31 cm ± 0.76, for the RG and LG groups, respectively.

Mortality rate 20 publications reported mortality rate with homogeneity test *I*^2 ^= 0%, *P *> 0.05, and analysis using a fixed effect model showed no statistically significant difference in mortality rate between the two groups (*OR *= 1.16, 95% *CI *= 0.76–1.76, *P *> 0.05) (Supplementary Figure 7). The average mortality rate was 1.32 in RG group and 3 in LG group.

Conversion rate 14 publications reported conversion rate with homogeneity test *I*^2 ^= 0%, *P *> 0.05, and a fixed effect model analysis showed no statistically significant difference in conversion rate between the two groups (*OR *= 0.64, 95% *CI *= 0.40–1.00, *P *> 0.05) (Supplementary Figure 8). The average conversion rate was 0.88 in RG group and 3.03 in LG group.

**Figure 8:**
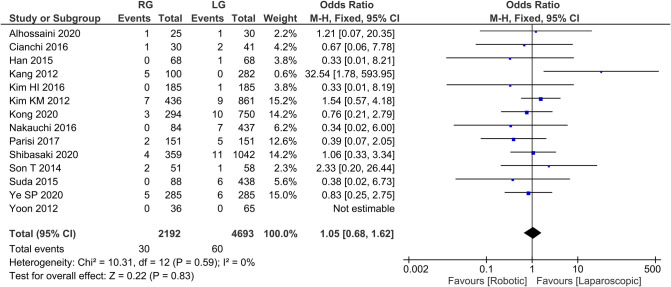
Comparison of reoperation rate between RG and LG group.

Reoperation rate 13 publications reported reoperation rate with homogeneity test *I*^2 ^= 0%, *P *> 0.05, and a fixed effect model analysis showed no statistically significant difference in reoperation rate between the two groups (*OR *= 1.05, 95% *CI *= 0.68–1.62, *P *> 0.05) (Figure 8). The average reoperation rate was 2.14 in RG group and 4.28 in LG group.

**Figure 9:**
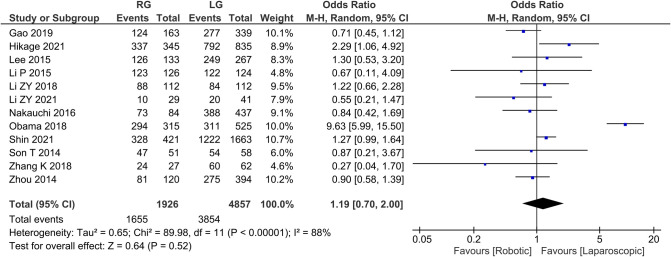
Comparison of 3-year survival rate between RG and LG group.

Overall survival 12 publications reported 3-year survival rates with homogeneity test *I*^2 ^= 88%, *P* < 0.05, and a random effects model analysis showed no statistically significant difference in 3-year survival between the two groups (*OR *= 1.19, 95% *CI *= 0.70–2.00, *P *> 0.05) (Figure 9). The average overall survival was 137.91 in RG group and 321.16 in LG group.

### Heterogeneity and Sensitivity Analysis

Heterogeneity is considered to be significant when *I*^2^ > 50% and *P* < 0.05. Our results suggest that there was heterogeneity in the time to first flatus, proximal margin, distal margin, tumor size, and overall survival (*I*^2^ > 50%, *P* > 0.05) (Table 3). Furthermore, substantial heterogeneity was also in operation time, intraoperative bleeding loss, lymph node dissection, and time to first food intake (*I*^2^ > 50%, *P* < 0.05) (Table 3). According to the MINORS score, high-quality literature with a score of more than 18 points was selected for sensitivity analysis.

### Publication of Bias

Evaluation of publication bias was accomplished using a funnel plot of intraoperative blood loss, lymph node dissection, and postoperative complications. There was no evidence of publication bias in the bilaterally symmetrical funnel plots of overall complications (Supplementary Figures 9, 10, 11).

## Discussion

In most cases of gastric cancer, gastrostomies are the mainstay of treatment. Almost thirty years ago, minimally invasive gastrostomies were introduced to reduce patient burden. As a result of the increasing availability of surgical robots, a robotic-assisted gastrectomy was performed for the first time in Japan in 2002 (52). Currently, robotic surgery is widely used in general surgery as well as other applications (22). In comparison to laparoscopic gastrectomy (LG), the feasibility and safety of the robotic-assisted (RG) technique were explored in this study.

Robotic surgery has become increasingly popular in a variety of surgeries due to its increased surgical precision and safety. Since the earliest application of robotics in surgery, it has evolved in five distinct categories: endoscopic, stereotactic, bioinspired, millimeter-scale microbots, and autonomous systems. Robotic surgery has shown to have dramatically superior clinical results when compared to laprascopic and open surgical techniques. In our study, of the 48 publications examined, 38 researches employed the Da Vinci surgical systems while the other 10 did not specify the surgical systems used (supplementary Figure 12.) According to the results of the comparative analysis of RG and LG gastric cancer treatment found in this study, there are disparities in efficacy between these treatments.

Based on the meta-analysis, it was found that RG requires a longer surgical procedure time than LG. One of the main reasons is that the robotic surgical system necessitates machine assembly at the beginning of the operation, and Jiménez-Rodrguez *et al.* (53) reported that the average preparation time for RG was 62.9% ± 24.6%min, but with experience the preparation time gradually decreases. Huang *et al.* (14) reported that the preparation time could be reduced to thirty minutes after 25 surgical operations. A study by Kang *et al.* (5) reported that the experienced RG group had a considerably shorter mean operation time than the inexperienced RG group. Furthermore, robotic surgery is a relatively new minimally invasive procedure that necessitates a learning process to master which is significantly shorter than LG. As reported by Mege *et al.* (54) and Huang *et al.* (14), the learning curve for LG surgery ranges from 30 to 50 cases, whereas surgeons performing 10–20 RG cases would accomplish a stable level of operative time. Huang *et al.* (14) compared LG to RG in the middle and later stages of the learning curve, finding LG to have a longer operative time than RG regardless of the stage. Consequently, once the learning curve is passed, the time spent intraoperatively in RG would be shorter than that in LG.

This meta-analysis revealed that the intraoperative blood loss in RG was less than that in LG, and the number of lymph nodes dissected in RG was higher than that in LG. There are abundant blood vessels and lymphatic vessels in the perigastric tissue. The process of LG perigastric tissue separation and lymph node dissection is prone to haemorrhage, which may affect the operator's ability to identify the tissue structure and to view the operation field. Due to the advantages of the robotic, these issues have been resolved (3–30), such as: (1) jitter filtering, the robotic arm eliminates the natural tremor in the human hand and improves the stability of the operation; (2) High-definition three-dimensional images, 3D three-dimensional images magnify the surgical field by 10–15 times, revealing the small blood vessels and tissue structure around the stomach, making the blood vessels around the stomach more secure, and improving the accuracy of the procedure; (3) Robotic arms have seven degrees of freedom to simulate the mechanical wrist, which allows for greater flexibility of operation and the ability to work in confined spaces; (4) The operator controls the robotic arm individually, eliminating the problem of incompatibility between the mirror arm and the operator; (5) The operator adopts a sitting position that provides both physical comfort and improves the efficiency of his or her operation; (6) Remote control by the operator so to avoid direct contact with the patient; (7) Reconstruction of the digestive tract to achieve a full endoscopic anastomosis which is suitable for obesity, barrel chests, high esophageal cut planes, a small costal arch angle, and anterior and posterior abdominal walls. There are several advantages to total endoscopic *in vivo* anastomosis for patients with the same diameter and width. These attributes, without a doubt, improve surgical precision and stability, minimise mistake rates, and promote minimally invasive surgery.

A patient's prognosis and degree of surgical cure are affected by the number of lymph nodes dissected at the time of surgery for early gastric cancer. As a treatment for intermediate and advanced gastric cancer, D2 lymph node dissection remains the standard procedure. Nonetheless, it is difficult to dissect D2 lymph nodes in LG. The included studies (3, 8, 22, 24, 28) showed that more lymph nodes had been cleared in the RG group than in the LG group, while the remaining studies showed no significant differences between the two groups in terms of lymph node clearance (6, 7, 11, 14, 17, 55). Across the included studies (3–30), the number of surgically cleared lymph nodes in RG ranged between 13.6 and 91.7, while all were able to reach the range of clearance of D2 lymph nodes. It has been revealed that RG can have therapeutic benefits that are comparable to LG and may even exceed them (for example in dissection, abdominal reduction, suturing *etc*).

We found that the RG group had a shorter first postoperative food intake period than the LG group. We found substantial discrepancies between Kim et al. (8) and Zhang et al. (10) among the independent literature examined. Possible reasons are (8, 10): (1) The robotic arm moves stably and flexibly during RG operation, helping to avoid overstretching and separation of tissues and accidental injury to blood vessels, thus causing less trauma to patients; (2) Adopting the concept of enhanced recovery surgery after perioperative management, Zhang *et al.* (10) reported earlier postoperative time to get out of bed, first gassing and eating time in the RG group compared to the LG group. As a result of the meta-analysis, however, the potential factor could not be the cause of the different postoperative hospital stay between the groups of RG and LG. A slight statistically significant difference was found between the two groups in terms of hospital stay, but the RG did appear to be preferred.

A meta-analysis of the data revealed that there was no difference in the rest of the data between the RG and LG group. Despite this, there is heterogeneity in first postoperative flatus time, postoperative complications, proximal incision margin distance, distal incision margin distance, tumor size, and 3-year survival rate. There may be a variety of reasons for this: (1) The operators included in the study may be in different stages of their RG development, and the indicators are heterogeneous. (2) The tumor size, location, and stage of included studies are different; (3) Preoperative management and surgical methods are also different, contributing to varying results. The findings of a high-quality non-randomized controlled trial, however, were also convincing when evaluating the short-term effects of surgery, as shown by Abraham *et al.* (56). After reviewing the high-quality literature, it was discovered that there was no significant difference between the two groups in terms of the number of lymph nodes dissected (WMD = 1.87, 95% CI = −1.24, 3.97, *P* > 0.05), and the rest of the results remained unchanged, indicating that systematic analysis results are relatively reliable.

This study has some limitations (1) the inclusion of the most recent literature and exclusion of studies with duplicate cases; (2) the inclusion of a relatively large number of studies, which increased the number of relevant cases; and (3) the systematic analysis of long-term survival information, such as the 3-year survival rate. Several limitations exist in this meta-analysis, including: (1) the included literature was retrospective, lacking high-quality randomized controlled trials, some of which had a small number of patients, which may have contributed to publication bias, and (2) the recurrence rate was not examined.

## Conclusion

Based on our meta-analysis, RG appears to have superior therapeutic effects than traditional LG for treating gastric cancer and both are safe and practical. Its future application opportunities will improve as more experience is gathered. In the future, large-scale, multi-sampled multicenter randomised controlled studies will be required to increase the reliability of RG in clinical therapy.
