# Supplementary figures and images for: Robotic Versus Laparoscopic Gastrectomy for Gastric Cancer: A Mega Meta-Analysis
Source: Front Surg. 2022 Jun 28;9:895976. doi: 10.3389/fsurg.2022.895976 (PMC9273891; doi:10.3389/fsurg.2022.895976)

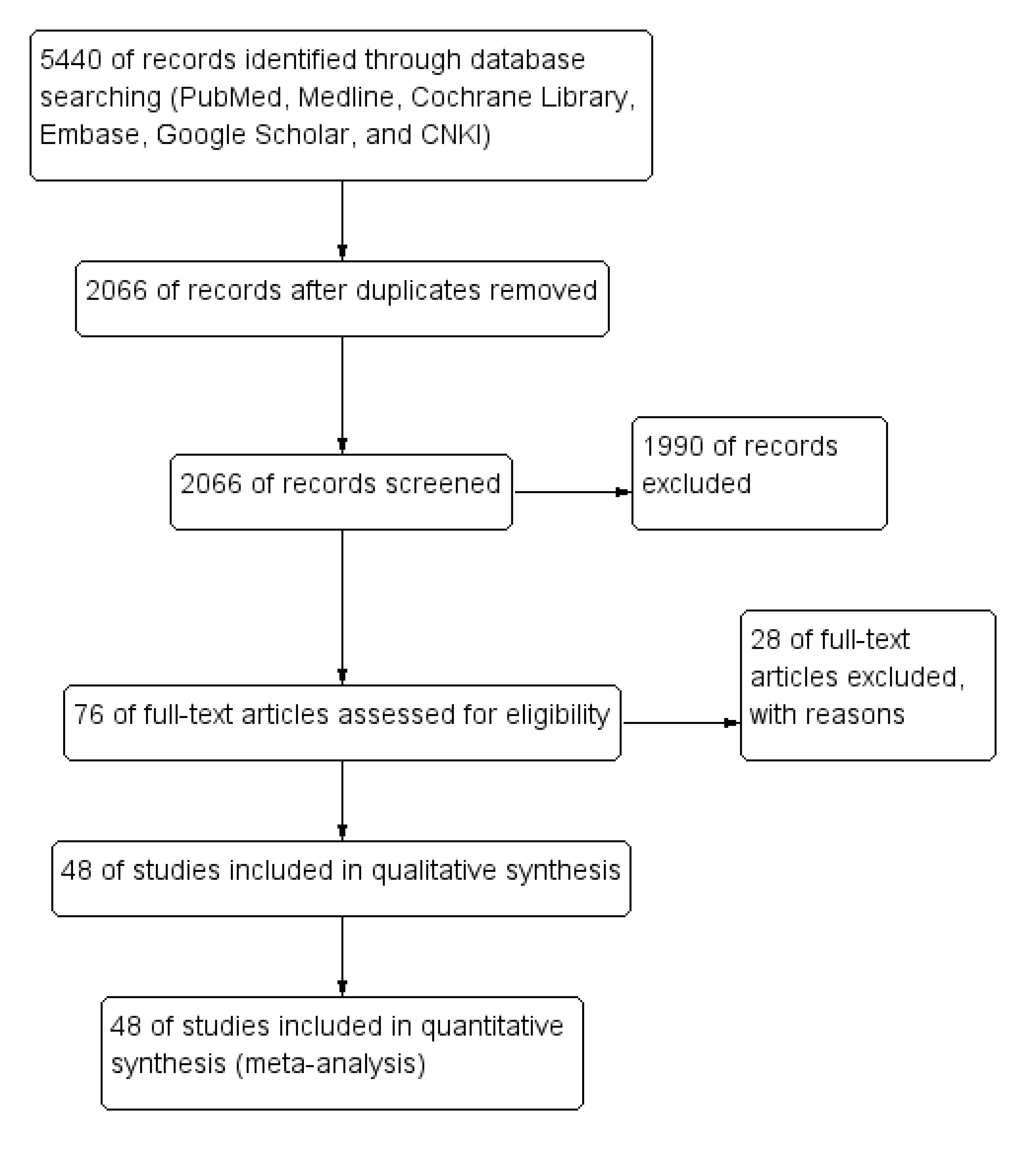

Supplement: Supplementary file 1 [file Image_1_v1.tif]

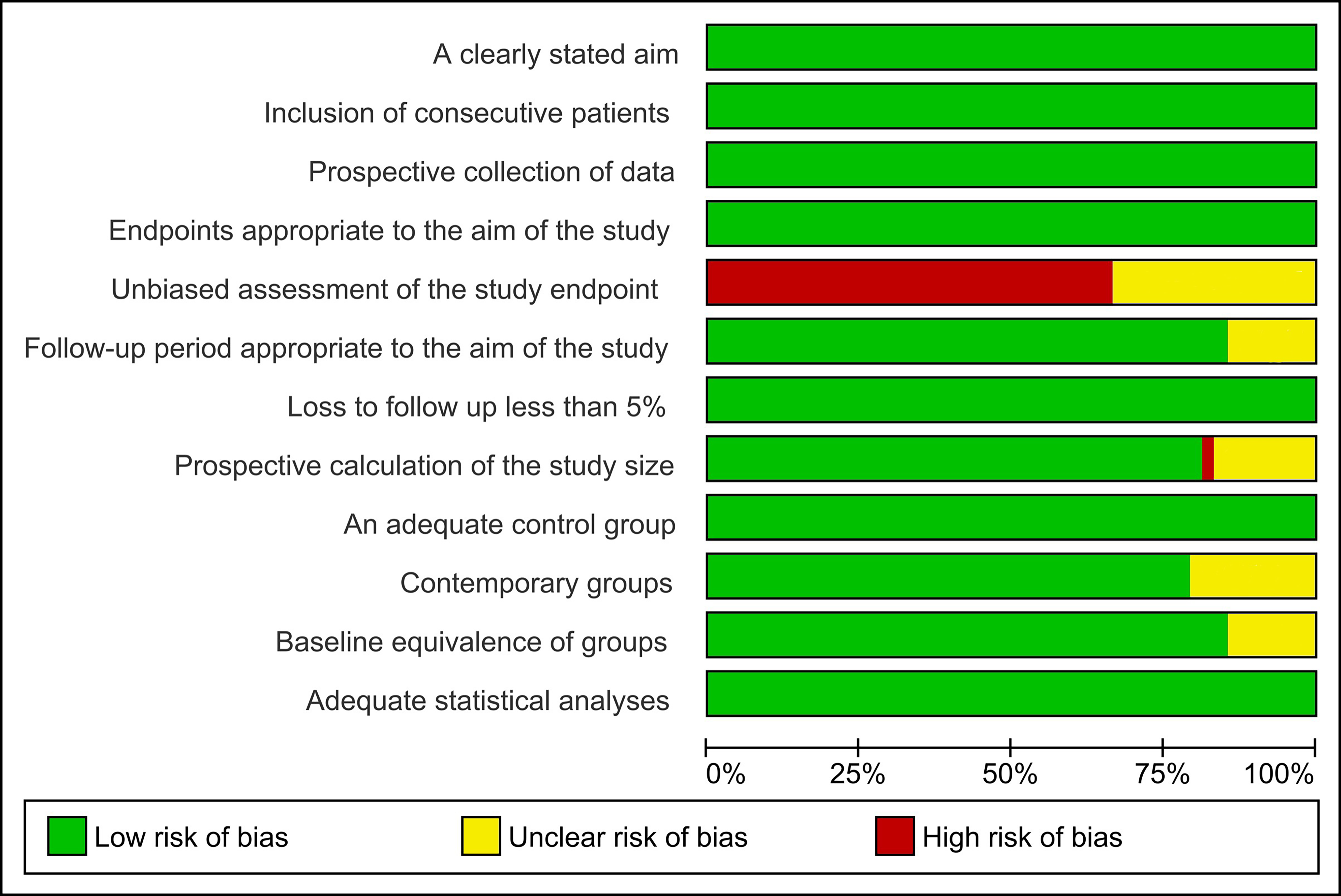

Supplement: Supplementary file 2 [file Image_2_v1.tif]

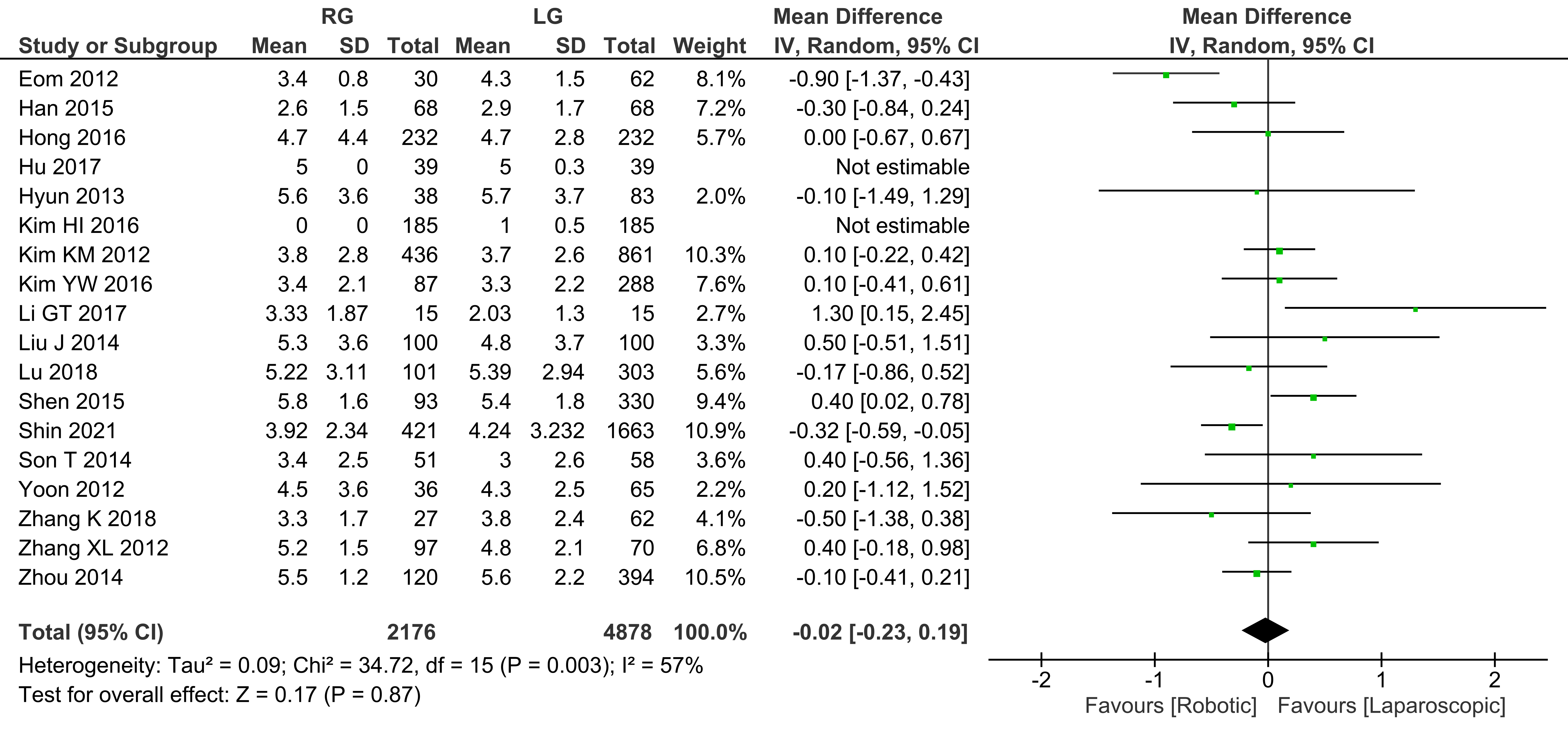

Supplement: Supplementary file 3 [file Image_3_v1.tif]

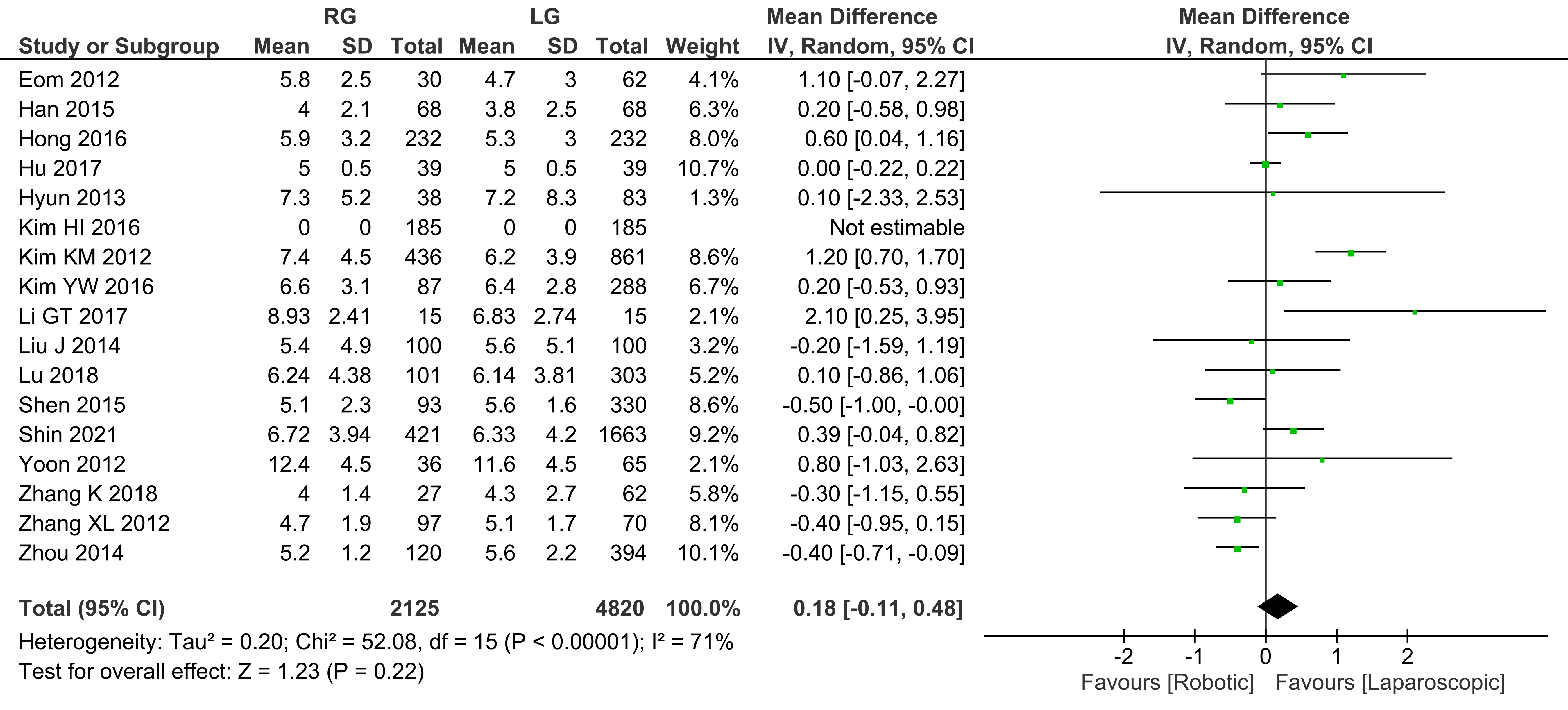

Supplement: Supplementary file 4 [file Image_4_v1.tif]

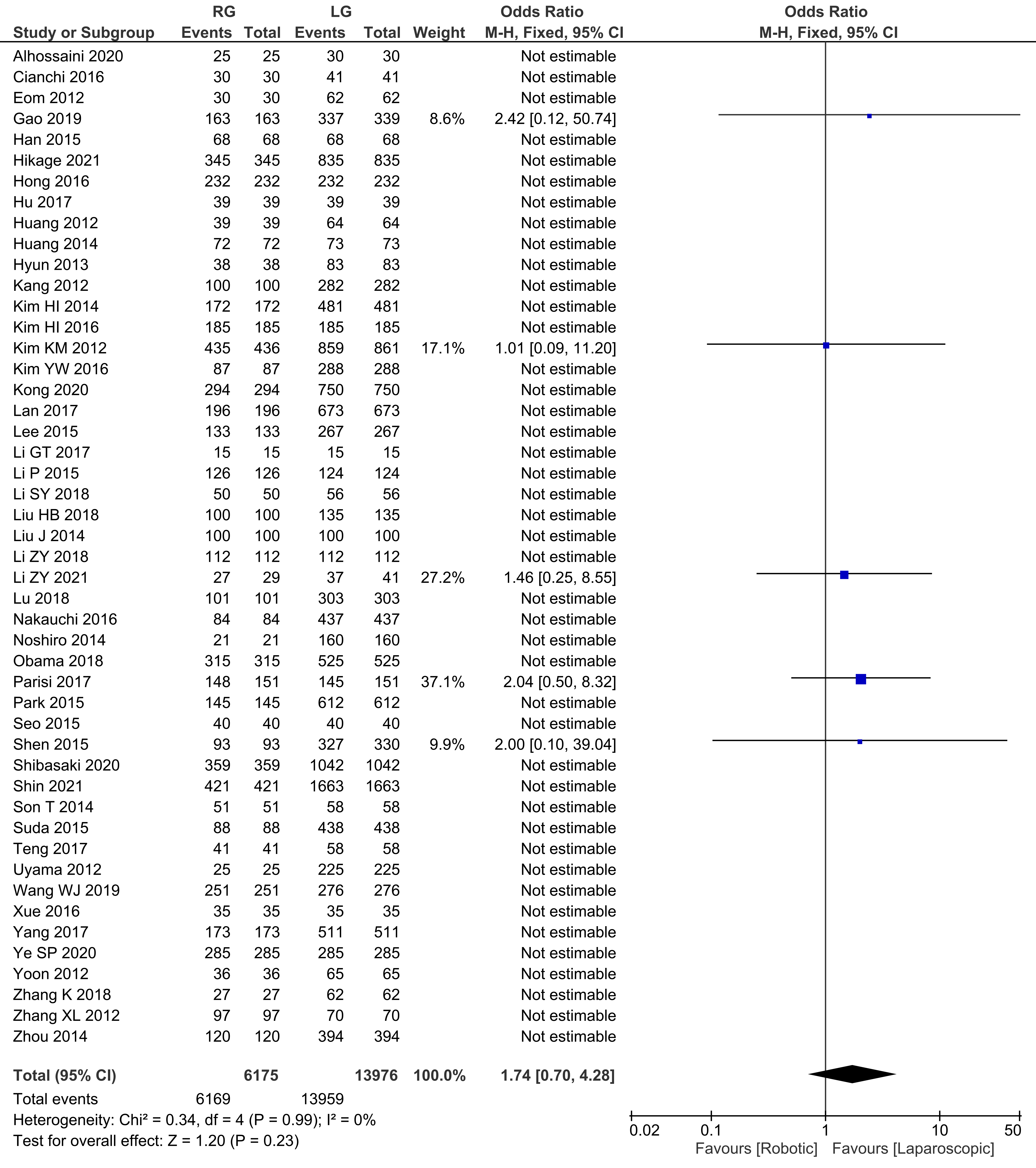

Supplement: Supplementary file 5 [file Image_5_v1.tif]

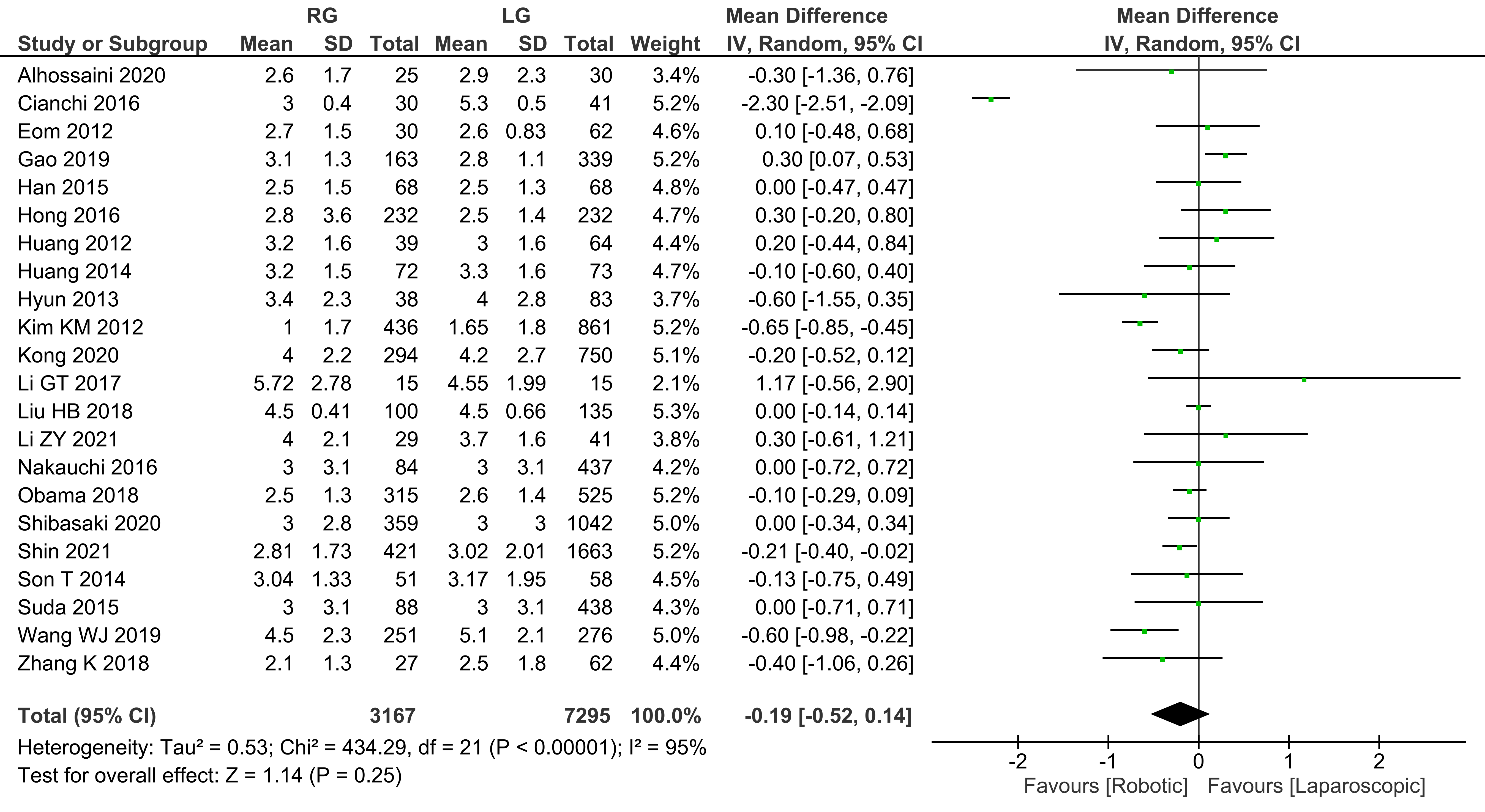

Supplement: Supplementary file 6 [file Image_6_v1.tif]

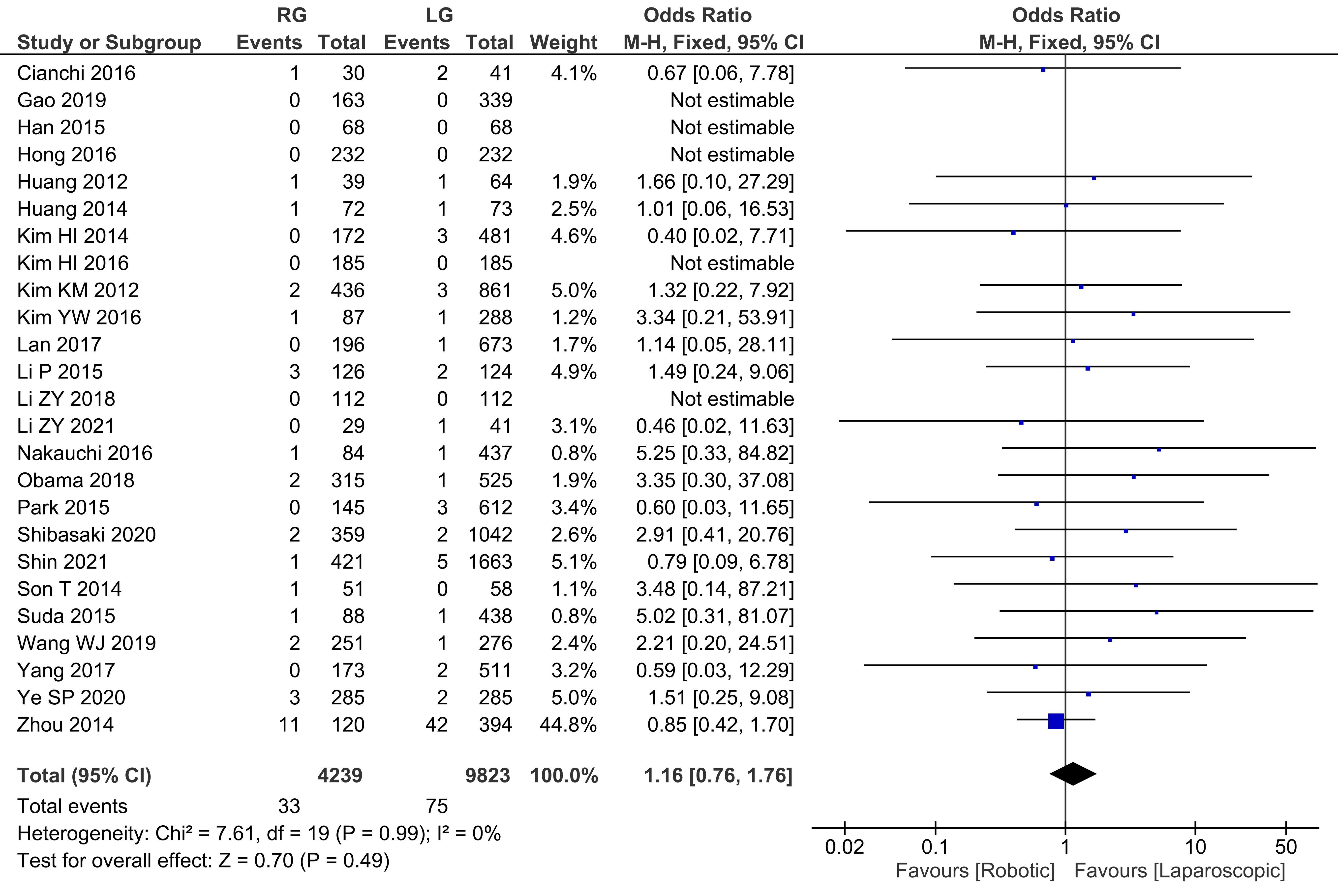

Supplement: Supplementary file 7 [file Image_7_v1.tif]

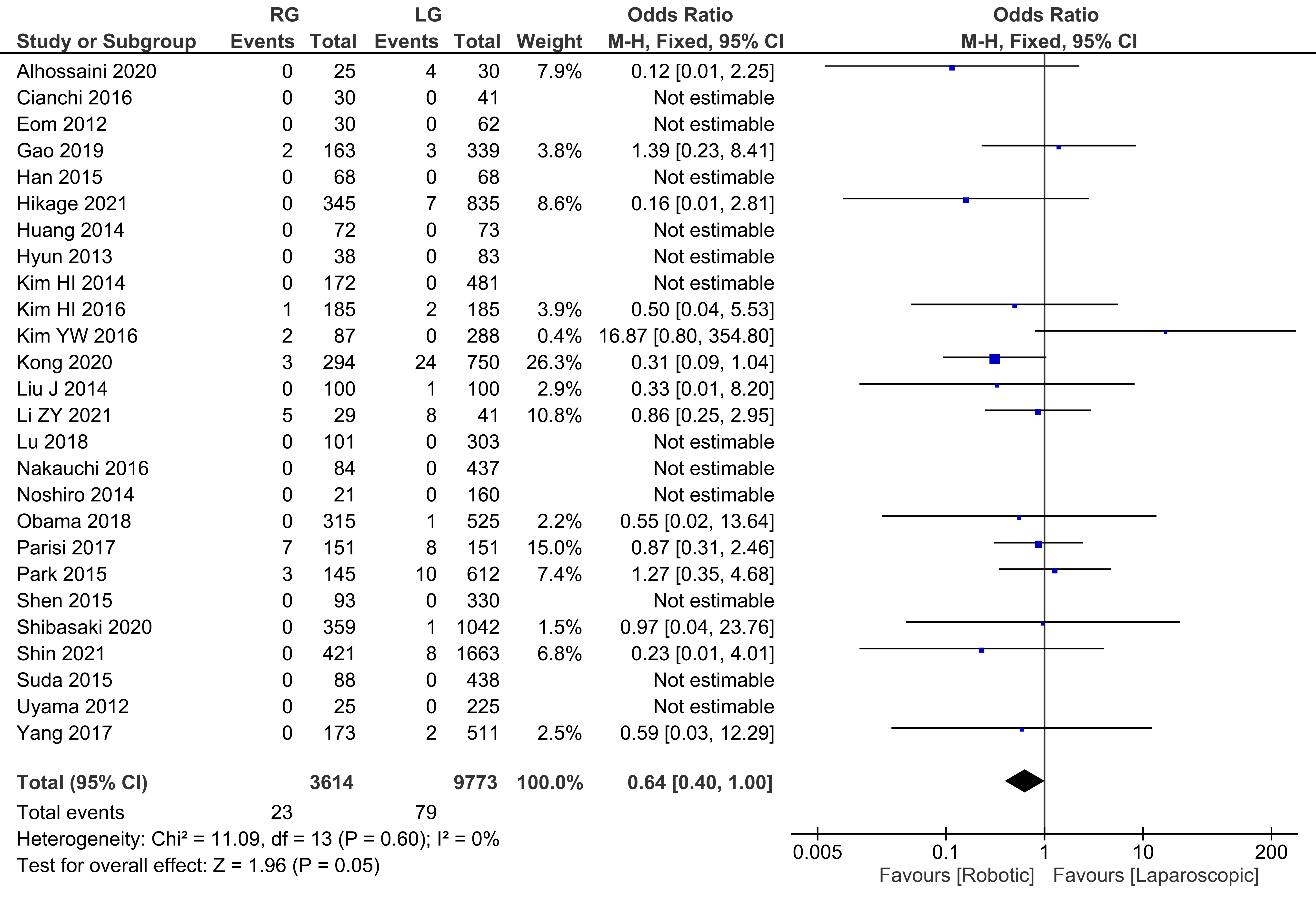

Supplement: Supplementary file 8 [file Image_8_v1.tif]

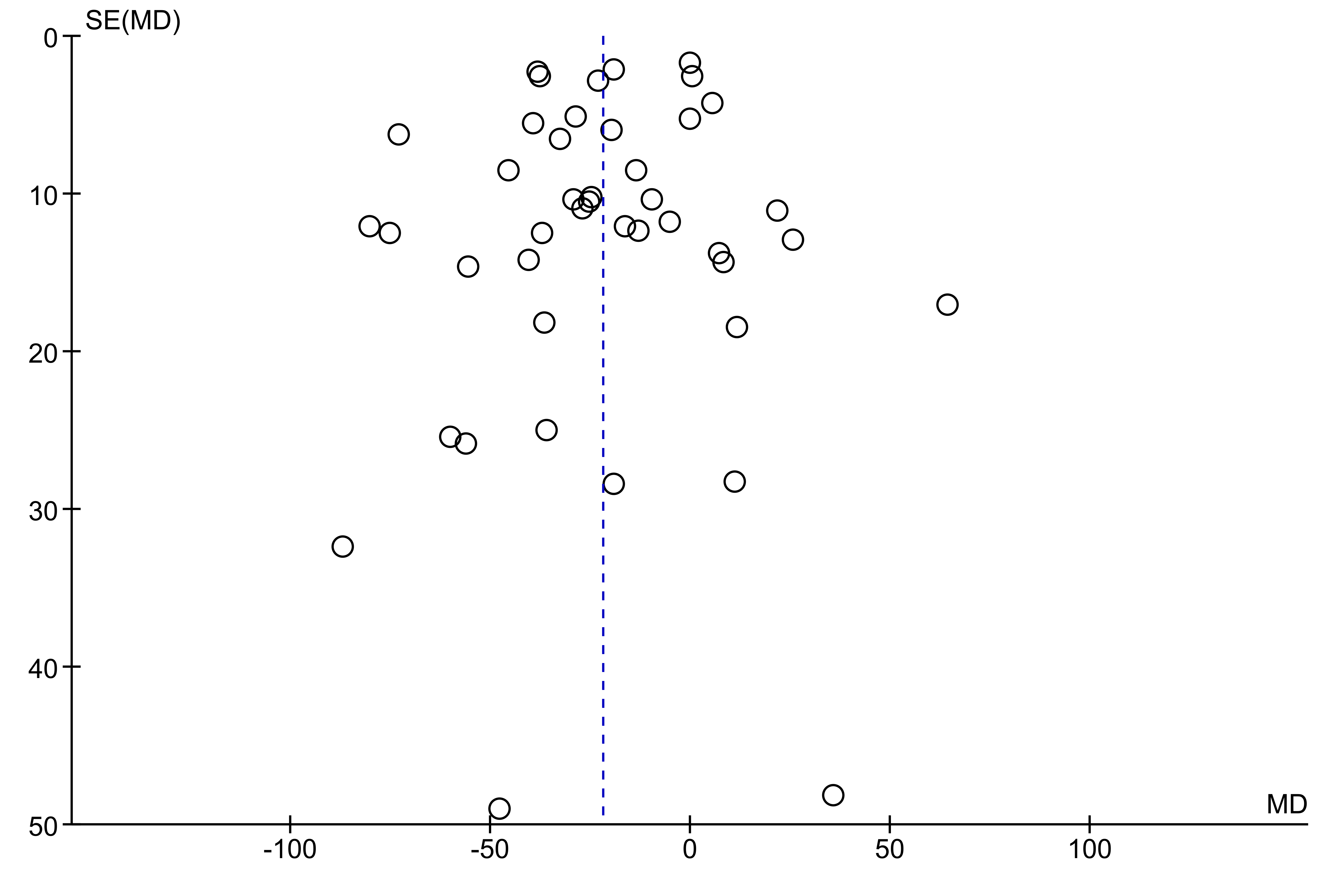

Supplement: Supplementary file 9 [file Image_9_v1.tif]

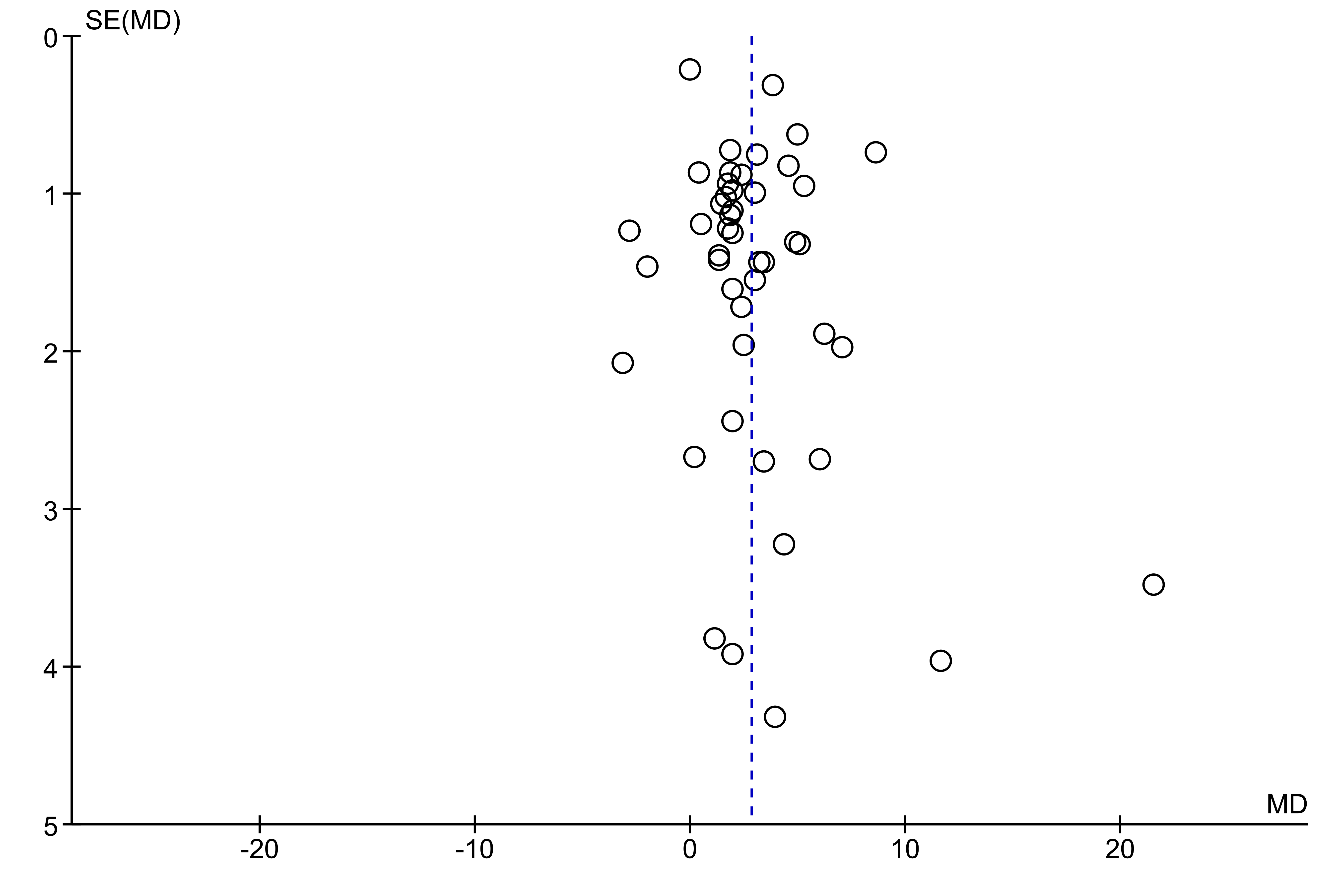

Supplement: Supplementary file 10 [file Image_10_v1.tif]

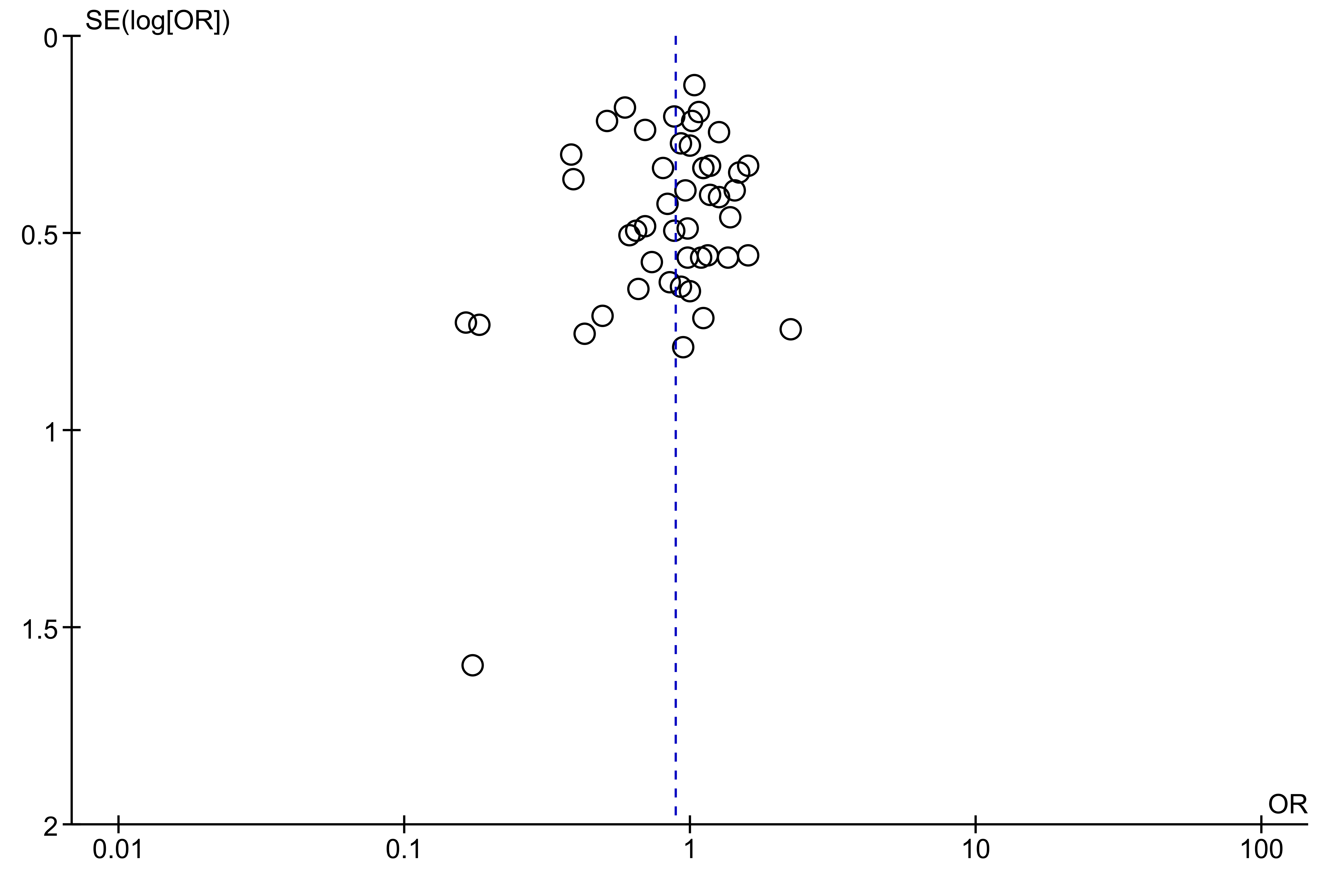

Supplement: Supplementary file 11 [file Image_11_v1.tif]

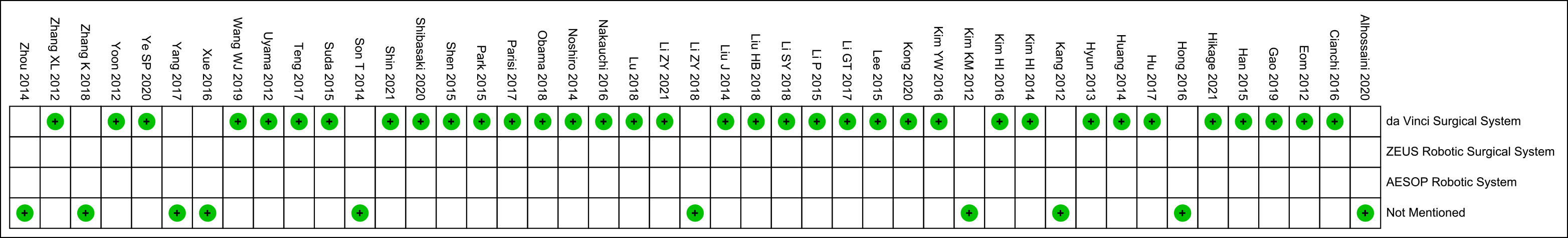

Supplement: Supplementary file 12 [file Image_12_v1.tif]
